# Cell-Based Therapies for Diabetic Complications

**DOI:** 10.1155/2012/872504

**Published:** 2011-06-09

**Authors:** Stella Bernardi, Giovanni Maria Severini, Giorgio Zauli, Paola Secchiero

**Affiliations:** ^1^Department of Morphology and Embriology and LTTA Centre, University of Ferrara, 44100 Ferrara, Italy; ^2^Baker IDI, Heart & Diabetes Institute, Melbourne, VIC 3004, Australia; ^3^Institute for Maternal and Child Health, IRCCS Burlo Garofolo, Trieste, 34137, Italy

## Abstract

In recent years, accumulating experimental evidence supports the notion that diabetic patients may greatly benefit from cell-based therapies, which include the use of adult stem and/or progenitor cells. In particular, mesenchymal stem cells and the circulating pool of endothelial progenitor cells have so far been the most studied populations of cells proposed for the treatment of vascular complications affecting diabetic patients. We review the evidence supporting their use in this setting, the therapeutic benefits that these cells have shown so far as well as the challenges that cell-based therapies in diabetic complications put out.

## 1. Introduction

The worldwide increase in the prevalence of diabetes mellitus reinforces the search for solutions to prevent it as well as to oppose the development and the progression of its complications. Particularly, the increasing prevalence of diabetes mellitus (DM) now affects adolescents and younger adults, thus promoting an earlier development of invalidating chronic diseases [1]. Experimental evidence suggests that cell-based therapies might represent a new and promising strategy for the treatment of diabetic vascular complications, and growing interest has recently been focused on mesenchymal stem cells and endothelial progenitor cells. Both cells types not only act against the mechanisms underlying diabetic complications but also rescue the abnormalities that stem cells present in diabetic patients, which contribute to the vascular complications. Notably, these cells avoid the ethical issues relating to the use of the embryonic cells. However, there are concerns about how the diabetic environment affects these cells. So, additional challenges for these cells include making them resistant to the diabetic environment and thus increasing their clinical efficacy [2]. 

On these premises, we will here review the evidence suggesting why adult stem/progenitor cells should be used in diabetic patients, the therapeutic benefits that these cells seem to offer for treating macrovascular and microvascular complications, and the challenges that cell-based therapies in DM present. 

## 2. Stem Cells

Adult stem cells comprise of roughly 3 different groups: the bone marrow stem cells (BM-SC), the circulating pool of stem/progenitor cells (which are also derived from the bone marrow), and the tissue-resident stem cells. BM-SC can be further categorized into multipotent adult progenitor cells, mesenchymal stem cells (MSC), and hematopoietic stem cells. The circulating pool of stem/progenitor cells includes different types of cells, among which the most studied for the setting of vascular complications are the endothelial progenitor cells (EPC). EPC were identified by Asahara et al. [3] in the search for circulating angiogenic cells. They observed that these cells were able to form new blood vessels and promote neovascularisation after ischemia.  Therefore, these cells seem to be the most promising in the setting of DM because of their potential utility in therapeutic neovascularisation and vascular repair. This paper will be focused on MSC and EPC, since these subsets of cells are the most studied in the field of the cell-based therapies for DM and for diabetic complications. 

MSC are a subset of cells that express on their surface CD54/CD102 (intracellular adhesion molecule), CD166 (vascular cell adhesion molecule), CD49 (*α*-integrin) as well as CD73 (5′ ribonucleotide phosphohydrolase) and CD90 which also regulate cell-to-cell interactions. They also express CD44 (receptor for hyaluronic acid), CD105 (modulator of cellular responses to TGF-*β*), and MHC1, whereas they do not express CD34, CD14, CD45, CD11a/LFA-1, and CD31, which are surface markers featuring hematopoietic cells and/or EPC instead [4]. MSC are present in the bone marrow, but can also be found in many other fetal and adult tissues. Indeed, they are generally isolated from bone marrow, adipose tissue, umbilical cord blood, and compact bone. MSC display a great therapeutic potential because, beyond their capability to differentiate into muscle, neural precursors, cardiomyocytes, and other cells types, they are able to migrate and home in injured sites, where they act both by regenerating tissues and by secreting trophic factors and paracrine mediators. Moreover, these cells interact with the immune system, particularly with dendritic cells, T cells and NK cells and therefore they modulate the outcome of immune cells responses, apparently by inhibiting TNF-*α* and INF-*γ* and by increasing IL-10 [5]. Therefore, their unique immunomodulatory properties make these cells appropriate for both autologous and allogenic transplants, since they avoid and/or actively suppress the immunological responses that cause rejection of transplants. For the same reason, they are now being studied for the treatment of immunological diseases, among which is type 1 DM [6]. Indeed, in the non obese diabetic mice “NOD mice”, the injection of MSC reduced the capacity of diabetogenic T cells to infiltrate pancreatic islets, thus preventing *β*-cell destruction [7]. Another model of type 1 DM is injecting mice with streptozotocin, which is a drug destroying the *β*-cells [8]. Also in this model, MSC were able to differentiate into insulin-producing cells releasing insulin in a glucose-dependent manner and improving the natural history of diabetes [9, 10]. Moreover, it has been demonstrated that, when cotransplanted with islets, MSC improved graft morphology and function by the promotion of revascularization [11].

EPC are adult hemangioblast-derived cells [12], which are characterized by the expression of CD34, vascular endothelial growth factor receptor 2 (VEGFR-2), and CD133, which has been included as marker expressed on primitive cells but not on differentiated ones. In fact, as the hemangioblasts destined to become endothelial cells differentiate, they downregulate the hematopoietic cells marker CD133^+^ (AC133) expression [12]. EPC can be isolated from human peripheral or umbilical cord blood and can also be found in bone marrow niches. The interest in EPC comes from the fact that these cells have been shown to have direct angiogenic actions and/or to be able to support angiogenesis. Particularly, like for MSC, part of their therapeutic potential could be related to their ability to secrete paracrine mediators. In this respect, several studies have shown that these cells release interleukins, growth factors, and chemokines that altogether regulate CD14^+^ cells, accelerate vascular network formation, and enhance healing processes [2]. Therefore, they are a promising therapeutic tool in the setting of diabetic complications, which are a consequence of dysfunctional vascular responses. 

## 3. Rationale for the Use of Adult Stem/Progenitor Cells for Diabetic Complications

Diabetic patients exhibit impaired mobilization of adult stem cells from the bone marrow [13] and dysfunctional circulating progenitor cells [14, 15]. A growing body of evidence has demonstrated that DM is associated with a generalized reduction in circulating EPC and that this decline is linearly correlated with the severity of DM, in terms of HbA1c and blood glucose, whereas it is inversely related to glucose control [16–18]. Busik and colleagues suggested that diabetic neuropathy, altering the circadian rhythm of bone marrow cells release, could be one of the factors accounting for the defective mobilization of stem/progenitor cells coupled to an increased number of cells trapped in the bone marrow [19]. Apart from diabetic neuropathy, the factors that have been classically related to impaired stem/progenitor cells mobilization are the direct and/or indirect effects of hyperglycemia. Fadini and colleagues have demonstrated that the bone marrow mobilization of cells is sensitive to hyperglycemia [13]. Using a model of hind limbs ischemia-reperfusion (I/R) injury for the study of EPC mobilisation in type 1 DM, they observed that diabetic rats were completely unable to mobilise EPC after I/R injury, compared to the control rats showing a mobilisation curve within 7 days after injury. However, after insulin administration and premedication with granulocyte-colony stimulating factor (G-CSF) and other stem cells factors, they achieved a partial recovery in postischemic EPC mobilisation [13]. This study suggests that mobilization mechanism is sensitive to chronic hyperglycemia and early on remains reversible. 

One of the mechanisms involved in the toxic effects of hyperglycemia on BM-SC seems to be the unbalance between nitric oxide (NO) and reactive oxygen species (ROS) [20]. It is known that hyperglycemia increases ROS formation which, by reacting with NO, lead to a reduction in NO bioavailability, therefore impairing NO signalling. Moreover, diabetic BM-SC display uncoupled endothelial NO synthase (eNOS) activity, promoting the production of ROS and so increasing the unbalance between ROS and NO [20]. Any reduction in NO bioavailability is believed crucial for BM-SC mobilization since NO-mediated signalling is essential for activation of MMP-9 which, in turn, shifts resident cells from a quiescent to a proliferative state and stimulates their rapid mobilization into the circulation [21]. Consistent with this concept, Segal and colleagues showed that incubating diabetic CD34^+^ cells with NO donors corrected their migratory defect, proving that impaired NO signalling in DM significantly contributes to bone marrow dysfunctional responses [22]. It is reasonable to suggest that MSC migratory properties could also be affected in DM. Diabetic patients display increased circulating levels of osteoprotegerin (OPG) [23], which is a soluble TNF-receptor with atherogenic [24] and diabetogenic [25] actions. Notably this peptide is the decoy receptor for the TNF-related apoptosis-inducing ligand (TRAIL) and displays antiatherosclerotic and antidiabetogenic properties. Our group has recently shown that TRAIL is able to promote the migration of BM-MSC *in vitro* [26]. OPG dose dependently neutralizes the promigratory activity of TRAIL [27], so the high levels of OPG observed in diabetic patients might impair the pro-migratory signalling driven by TRAIL, accounting for the abnormalities of BM-SC in DM. 

Several *in vitro* works have pointed out that the diabetic *milieu* does not only impair BM-SC mobilization, but it also affects the lifespan and the functions of adult stem cells which may account for the reduction in circulating EPC. Particularly, hyperglycemia has been shown on its own to accelerate the senescence of EPC by the activation of p38/MAPK [28] and Akt/p53/p21 [29] pathways or by downregulation of sirtuin 1 [30]. In this setting, the senescence of EPC could also be due either to the NO reduced bioavailability mentioned previously, since it has been demonstrated that NO delays endothelial cells senescence through the activation of telomerase [31], or to the increased apoptosis induced by ROS. It has indeed been demonstrated that the deletion of p66ShcA, which is a gene regulating the apoptotic responses to oxidative stress, rescues the EPC defects induced by hyperglycemia [32]. However, in a work aimed at defining cross-sectionally the time course of EPC alterations in type 2 DM and to identify potential mechanisms of progenitor cells reduction, Fadini and colleagues found that the lower the count of CD34^+^ cells the higher their apoptotic rate but also that there was no difference in the apoptotic rate between patients with and without DM and that the percentage of EPC apoptosis was too low to fully explain a decreased cell count [33]. Thus, *in vivo* studies have not confirmed yet if diabetic EPC have a shortened lifespan, and other mechanisms, apart from the reduced lifespan, may account for the reduction of these cells in DM. Likewise, when cultured in hyperglycemic conditions, MSC increase the production of intracellular ROS which reduce hypoxia-induced factor1*α* (HIF1*α*) expression and consequently attenuate hypoxia-induced vascular endothelial growth factor (VEGF)-A and platelet-derived growth factor (PDGF)-B transcription [34]. Moreover, it is well known that hyperglycemia leads to nonenzymatic glycosylation of proteins and subsequent formation of advanced glycation end products (AGEs) that interacting with their own receptor, RAGE, then activate several intracellular pathways ultimately leading to tissue damage [1]. In this setting, AGEs directly impair the reparative function of both EPC and MSC, and several works have evaluated AGEs deleterious effects on EPC [35–37] as well as on MSC. After isolation of MSC from rats with type 1 DM, Stolzing and colleagues studied their *ex vivo* ability to proliferate and differentiate into the fibroblastic colony-forming unit. They reported that colony size and number were significantly reduced in diabetic rats, mainly because of the induction of cell apoptosis and senescence by AGEs [38]. Consistent with this, when treated with glyceraldehydes and glycolaldehydes, MSC showed reduced cell proliferation, increased cell apoptosis, and impaired differentiation into adipogenic, chondrogenic, and osteogenic clones. These effects were partially prevented by the antiserum against RAGE [35, 39]. 

Altogether these experimental works demonstrate that DM affects the mobilization and the functions of adult stem cells; therefore they provide the rationale for the use of adult stem cells for diabetic complications. 

## 4. Adult Stem/Progenitor Cells for the Treatment of Macrovascular Complications and Diabetic Cardiomyopathy

### 4.1. Macrovascular Complications

Both type 1 and type 2 DM increase the incidence and progression of atherosclerosis [40] into large arteries and the development of macrovascular complications. Their major clinical manifestations are coronary artery disease (CAD), peripheral artery disease (PAD), and stroke. In particular, patients with DM have a 2–4 fold increased risk of fatal myocardial infarction, PAD and stroke, together with poorer long-term outcomes [40, 41]. The evidence supporting the utility of cell-based therapies in this setting, and particularly EPC-based therapies, comes from clinical studies showing an inverse relation between the number of EPC and the occurrence of cardiovascular diseases (CVD). Consistent with the reduction of CD133^+^cells observed in patients with CVD, CD34^+^/VEGFR-2^+^ and CD133^+^ cells counts have indeed been shown to predict the occurrence of CVD in one-year follow-up studies [42, 43], whilst CD34^+^ and CD34^+^/KDR^+^ cells counts might be helpful in stratifying the cardiovascular risk of the patients [44]. As expected, in patients with DM and metabolic syndrome, circulating CD34^+^ cell numbers were also found to be an independent risk marker of CVD [45], leading to the hypothesis that the reduction in circulating progenitors is not only a marker but also a causative factor for the increase in cardiovascular events. Interestingly, significantly lower numbers of EPC were observed in diabetic patients when PAD had developed [46].

**Figure 1:**
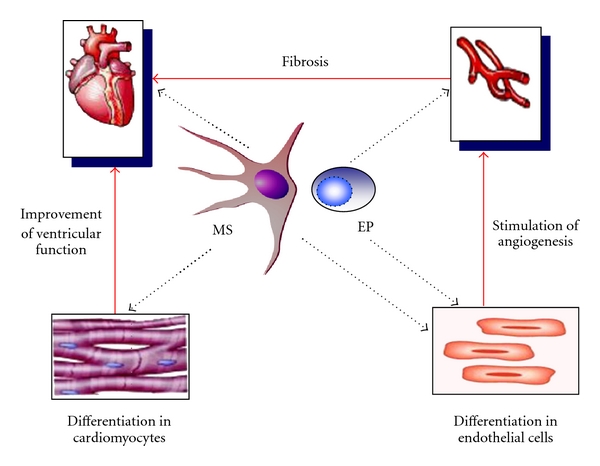
Effects of MSC and EPC on myocardial repair/regeneration and angiogenesis. The activities of MSC and EPC may derive from their differentiative ability (into cardiomyocytes and/or endothelial cells) as well as from secretion of paracrine mediators promoting myogenesis, angiogenesis, and heart functionality, in direct and/or indirect manners.

Although a study by Ma and colleagues showed that the treatment with EPC reduced the stenosis obtained after denudation of the common carotid artery in rabbits [47], data on the utility of cell-based therapies to prevent atherosclerosis are indeed conflicting. Silvestre and colleagues have demonstrated that transplantation of BM-SC in ischemic Apolipoprotein E-knockout mice, which is the most largely used animal model for the study of atherosclerosis [41], disappointingly accelerated atherosclerosis without altering the plaque composition [48]. Moreover, smooth muscle progenitor cells have been shown to contribute to the exaggerated intimal hyperplasia found in DM [49]. Consistent with this, in the clinical trials evaluating cell-based therapies after myocardial infarction, one of the major side effects that have been observed was the aggravation of the restenosis [50]. In this setting, another issue that needs to be further investigated is whether arrhythmias are a real safety concern, given that a higher number of arrhythmic events have been reported after intramyocardial delivery of cells, particularly skeletal myoblasts [51]. However, the trials aimed at myocardial repair in patients with acute myocardial infarction have also proven that the intracoronary infusion of BM-SC or CD133^+^or MSC is associated with an improvement in the global left ventricular ejection fraction, a reduction in the end-systolic left ventricular volumes, and a better perfusion in the areas of infarction [52, 53]. These effects are supposed to be due, at least in part, to the ability of these cells to stimulate myocardial repair/regeneration and neovascularisation (Figure 1).

Cell-based therapies appear promising also in the setting of PAD. A growing body of evidence strongly suggests the utility and effectiveness of adult stem cells for therapeutic neovascularisation both in absence [54–57] and in presence [58–60] of DM. Diabetic PAD is a systemic disease characterized by occlusion of peripheral arteries together with a severe impairment in the development of collateral vessels believed to be caused by endothelial dysfunction and the lack of growth factors, such as VEGF, both driven by glucotoxicity [1, 40]. The ability of EPC and MSC to produce angiogenic factors (by restoring the physiological levels of VEGF and HIF1*α*) and to differentiate into vascular cells in the periphery [61] has been implicated in the recovery of the native blood flow in ischemic hind limbs after their use. Recently, the transplantation of MSC for therapeutic neovascularisation has also been proven beneficial in type 1 diabetic patients with bilateral upper extremity digital gangrene, demonstrating improved arterial perfusion, good healing of all amputation sites, and cessation of pain [62]. 

Furthermore, in the context of macrovascular complications, intravenous autologous MSC transplantation has been shown to be able to reduce the mortality rate in patients with ischemic stroke [63]. 

### 4.2. Diabetic Cardiomyopathy

Diabetic cardiomyopathy should be considered separately from the so-called macrovascular complications of DM, since it corresponds to the stage characterized by the development of ventricular dysfunction in patients affected by DM, in the absence of CAD, valvular heart disease, or hypertension [64]. Its features, which are heterogeneous, are mainly due to cell apoptosis [64] associated with a dramatic reduction in tissue-resident stem cells [65], extensive myocardial fibrosis, and capillary rarefaction [66]. In particular, it has been shown that the abnormal myocardial matrix deposition associated with DM relies on increased collagen synthesis and on its reduced degradation, whose main effectors are the metalloproteases (MMP). Consistent with this, the diabetic myocardium is characterized by decreased activity of MMP-2, leading to increased collagen accumulation, and increased activity of the apoptotic factor MMP-9 which is responsible for apoptosis of endothelial cells, reduction of capillary density, and poor myocardial perfusion instead. In a study on rats with type 1 DM, the intravenous infusion of MSC improved cardiac function through increased angiogenesis and attenuated cardiac remodelling. Eight weeks after the induction of DM, rats were infused with MSC, which then homed into the myocardium and led to increased myocardial arteriolar density and decreased collagen content in the diabetic myocardium. Interestingly, increased MMP-2 activity and decreased transcriptional level of MMP9 were also reported [67]. However, even more fascinating is the possibility of developing noninvasive cell-based therapies relying on the trophic activities of MSC (Figure 1). A recent study with a hamster heart failure model has demonstrated that an intramuscular delivery of MSC would be sufficient to significantly improve ventricular function, enhancing capillary and myocyte densities, attenuating apoptosis, and reducing fibrosis. This was reported to be due to a trophic cross-talk among the injected MSC, the bone marrow, and the heart [68]. 

## 5. Adult Stem/Progenitor Cells for the Treatment of Microvascular Complications and Wound Healing

### 5.1. Diabetic Nephropathy

**Figure 2:**
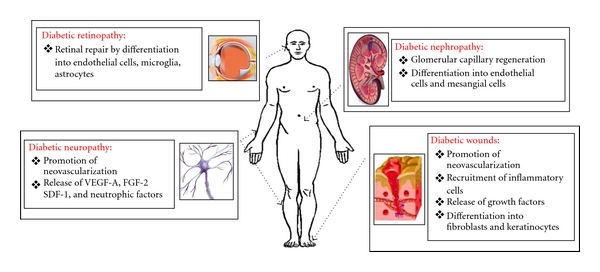
Potential role of EPC and MSC in the control of diabetic microvascular complications and wound healing. Diabetes mellitus is characterized by microvascular complications (retinopathy, nephropathy, and neuropathy) and prolonged/incomplete wound healing. Cell-based therapies may control these complications by different potential mechanisms.

Diabetic nephropathy is now the most common cause of end-stage renal failure in the Western societies. The arterial damages and the changes to the glomerular ultrastructure, mainly mesangial expansion and glomerular membrane thickening, are the principal mechanisms causing diabetic nephropathy [1]. These effects are both driven by hyperglycemia, and thus it is not surprising that one of the most important interventions in preventing diabetic nephropathy, or attenuating it, can be achieved by tight glycemic control [69]. In this setting, it has been shown that EPC mobilize into damaged glomeruli [70], possibly participating in glomerular capillary regeneration. More recently, a subset of hematopoietic stem cells, featured by the expression of the surface molecules CD24^+^/CD133^+^, has been shown promising as it was able to regenerate both tubular cells and podocytes. This is quite significant because the depletion of these cells plays a crucial role in the development of glomerulopathies which are now believed to be podocytopathies [71]. However, in the context of cell-therapy approaches for diabetic nephropathy, the most attractive candidates seem to be the MSC. So far, several works have shown that MSC administration can both prevent and treat diabetic nephropathy. In mice with type 1 DM [72], MSC had the ability to induce *β* pancreatic islets regeneration with consequent achievement of a better glycemic control that, in turn, prevented the development of diabetic nephropathy. MSC also had the ability to slow the progression of diabetic nephropathy through mechanisms independent from glycemic control [73] (Figure 2). Indeed, after an infusion of MSC, 11% of these cells engrafted into the kidneys, where they differentiated into endothelial cells and possibly mesangial cells. This was associated with a significant decrease in mesangial thickening, extracellular matrix deposition, and macrophages infiltration [74]. 

### 5.2. Diabetic Neuropathy

Diabetic neuropathy is estimated to affect over half of the patients with DM. It is a form of neuropathy that affects the somatic and autonomic divisions of the peripheral nervous system, but the spinal cord and the higher central nervous system can also be damaged. The main underlying cause is glucotoxicity and its downstream effects [1]. High glucose levels, oxidative stress, and AGEs reduce nerve blood flow and impair neurotrophic support, altogether leading to neural cells degeneration. Cell-based approaches promoting endogenous production of neurotrophic factors, such as nerve growth factor (NGF), hepatocyte growth factor (HGF), neurotrophin 3, or VEGF, have recently shown some success [1] (Figure 2). 

In the setting of diabetic neuropathy, both EPC and MSC have been investigated. Many studies have shown that transplantation of EPC ameliorates the blood flow to peripheral ischemic tissues. Naruse and colleagues investigated whether a unilateral intramuscular injection of EPC into the hind limb skeletal muscles could ameliorate diabetic neuropathy. After such transplantation, more vessels were observed in the injected sites, and this was associated with an amelioration of sciatic nerve blood flow and motor nerve conduction velocity. These data suggest that the ability of EPC to treat diabetic neuropathy is due to the promotion of therapeutic neovascularisation [75] (Figure 2). In addition, in another study, intramuscularly injected EPC not only increased nerve blood flow but also increased the number of vasa nervorum ameliorating the microvascular insufficiency typical of diabetic neuropathy [76]. As a matter of interest, in the same study, EPC were also found to increase the production of VEGF-A, brain-derived neurotrophic factor, fibroblast growth factor (bFGF)-2, stroma-derived factor-1 (SDF-1), and other neurotrophic factors, therefore indicating that EPC may reverse various manifestations of diabetic neuropathy through both angiogenic and neurotrophic properties (Figure 2). Consistent with this, *in vitro* studies proved that EPC were able to make Schwann and endothelial cells proliferate and to reduce the number of apoptotic cells [76].

Also MSC have displayed angiogenic and neurotrophic properties. Four weeks after their intramuscular injection, MSC started producing bFGF and VEGF, and this was associated with an increased ratio between capillaries and muscular fibers, an increased blood flow to the sciatic nerve, an improvement in motor nerve conduction velocity, and a reduced hyperalgesia [77]. Although one of the most exciting properties of MSC, from a therapeutic perspective, is their ability to differentiate into multiple cellular phenotypes, any differentiation into neural cells, such as astrocytes, oligodendrocytes, and Schwann cells, has not yet been observed [77] (Figure 2). 

### 5.3. Diabetic Retinopathy

DM is the leading cause of cases of blindness among adults. Diabetic retinopathy is characterized by a complex of vascular and retinal lesions, all ultimately due to hyperglycemia. This disorder can be categorized into the non proliferative diabetic retinopathy (NPDR) and the proliferative diabetic retinopathy (PDR) [1]. NPRD is characterized by vascular changes leading to retinal ischemia, whereas PDR is the result of an aberrant reactive neovascularisation. Most works carried out in this field have featured EPC, reporting a reduced number of EPC in patients with NPDR [78, 79] but an increased number of EPC in those with PDR. This is consistent with the hypothesis that, since EPC can rescue and maintain the existing retinal capillary bed in healthy patients, the reduced number of EPC observed in DM might predispose to NPDR [2]. Once the damage is initiated, an inflammatory reaction will take place and the bone marrow will respond by increasing the mobilization of EPC, which will eventually result in an abnormal neovascularisation leading to PDR. To date, the studies carried out on ischemic retinal injury have documented the participation of adult stem cells in the retinal repair, showing their ability to home into damaged areas and to differentiate into endothelial cells, microglia, and astrocytes [80–82] (Figure 2). However, these studies were all conducted in animal models of ischemic retinal injury and so concern still remains about the viability of these therapeutic options in the long run, since they could worsen the aberrant reactive neovascularisation featuring the PDR that follows any ischemical retinal injuries. Therefore, PDR may represent a contraindication for angiogenic cell-based therapies. 

### 5.4. Wound Healing

Another common complication of DM is represented by the prolonged and incomplete wound healing, caused by compromised angiogenesis, diminished cells recruitment, lack of growth factors, and impaired formation of collagen matrix. It has been demonstrated that generally the number of MSC increases considerably in the site of an injury, and that after a vascular trauma a rapid mobilization to the injured site of EPC also takes place. Wound healing normally results from a combined effort of inflammatory and noninflammatory cells recruited to the injured site. Recent studies suggest that MSC and EPC are a significant proportion of the noninflammatory cells that migrate to the skin. In DM, the number of EPC within the granulation tissue has been found to be significantly reduced with respect to non diabetic controls [83] and locally increased apoptosis and decreased proliferation of these cells have also been reported. Several works have shown that MSC accelerate wound closure by differentiating into fibroblasts and keratinocytes, and promoting neovascularisation and regeneration of appendages and recruiting inflammatory cells into wounds [84, 85] (Figure 2). Transplantation of EPC has also been shown to enhance wound healing in mice [86], and this seems to rely on the release of paracrine mediators, such as the release of VEGF, HGF, G-CSF, and PDGF [84, 87]. As expected, in the setting of DM, the same mechanisms, mentioned above for MSC and EPC, have been shown to enhance wound healing [88, 89] and to also be an effective treatment of foot ulcerations [90–92] (Figure 2). 

## 6. Genetic Manipulation and Pharmacological Strategies Aimed at Reversing the Alterations of Adult Stem/Progenitor Cells in Diabetes

### 6.1. Genetic Manipulation

The evidence obtained so far makes for a compelling argument for the use of MSC and/or EPC in the setting of DM [2]. Because of the broadly dysfunctional cell functions found in DM, it is believed that cells to be used for treatment of diabetic complications should be equipped with cellular and molecular tools to make them withstand the *in vivo* diabetic *milieu*. Thus, studies into the genetic modification and/or manipulation of diabetic cells have commenced as approaches in overcoming this issue. In recent work, Marrotte and colleagues [93] transfected EPC with the gene of manganese superoxide dismutase, in order to correct its decreased expression found in diabetic EPC. They found that, after this *ex vivo* manipulation, the EPC transplanted contributed significantly to the accelerated wound healing in a type 2 DM animal model. So far, several molecules have been targeted, such as human telomerase reverse transcriptase (hTERT), which was shown to delay EPC senescence [94], and the glycogen synthase kinase 3-*β*, which enhanced the EPC vasoregenerative potential [95]. In MSC, the overexpression of GATA-4, CXCR4, and Akt-1 led, respectively, to increased cell survival and angiogenesis [96], enhanced *in vivo* mobilization into ischemic areas [97], and better functional repair in a mouse infarct model [98]. Although the genetic manipulation of adult stem cells dysfunctions in DM has shown promising results, one should be very cautious when adopting this approach because of its potential side effects. For instance, targeting senescence/survival regulatory pathways warrants greater understanding given the risk of malignant transformation of the cells. 

### 6.2. Pharmacological Strategies

Other approaches have pharmacologically targeted the intracellular dysfunctions that take place in DM. For example, the effects of AVE9488 [99], GH [100], both stimulating eNOS, and those of rosiglitazone, which has antioxidant properties [101] have been studied as treatments for the reduced NO bioavailability [102]. Interestingly, Sorrentino and colleagues showed that the effect of rosiglitazone treatment was comparable to that of small-interfering RNA silencing NADPH oxidase subunit p47. Both approaches reduced NADPH oxidase activity, restoring NO bioavailability, and improved *in vivo* reendothelization capacity of EPC isolated from diabetic patients. However, whether increasing NO production and bioavailability may result in higher production of reactive oxygen species that will further increase oxidative stress leading to vascular damage is unknown yet. The blockade of p38/MAPK pathway, using its specific inhibitor SB203580, has also been assayed. Seeger and colleagues demonstrated that the *ex vivo* treatment of EPC with SB203580 was able to significantly ameliorate their revascularisation properties, possibly through the regulation of their proliferation and differentiation [103]. *Ex vivo* treatment of MSC with IGF-1 and IGF-2 made MSC regain the functions affected by DM [104]. Finally, antagonists of CXCR4 (such as AMD3100 and SDF-1*β*P2G), which disrupt the interaction between the CXCR4 receptor (on hematopoietic cells) and the CXCL12 (expressed by stromal cells), have already been shown promising in accelerating blood flow restoration in diabetic mice [105]. 

## 7. Conclusions

The past decade has provided new and fascinating *in vitro* and *in vivo* data supporting the use of MSC and EPC for the treatment of diabetic complications. However, among the issues raised, the possible contribution of these cells to lesion formation, in terms of atherogenesis, neointimal hyperplasia, and retinal aberrant angiogenesis, as well as the potential risk of their malignant transformation will certainly require further long-term analysis. Also, it is yet to define the best way to make these cells withstand the diabetic *milieu *in the long run. Therefore, a greater understanding of MSC and EPC biology, both in *in vitro* and *in vivo* studies, is needed to establish the safety of their use as a novel and efficient therapeutic agents in the treatment of complications of DM.
